# Pathophysiological and Genetic Aspects of Vascular Calcification

**DOI:** 10.1155/2020/5169069

**Published:** 2020-04-14

**Authors:** Luis Fernando Escobar Guzman, Cristian Andres Escobar Guzman, Neuza Helena Moreira Lopes

**Affiliations:** ^1^Heart Institute (InCor), University of São Paulo Medical School, São Paulo, Brazil; ^2^Department of Pathology, University of São Paulo Medical School, São Paulo, Brazil

## Abstract

Recent evidence suggests that vascular calcification is an independent cardiovascular risk factor (CRF) of morbidity and mortality. New studies point out the existence of a complex physiopathological mechanism that involves inflammation, oxidation, the release of chemical mediators, and genetic factors that promote the osteochondrogenic differentiation of vascular smooth muscle cells (VSMC). This review will evaluate the main mechanisms involved in the pathophysiology and genetics modulation of the process of vascular calcification. *Objective.* A systematic review of the pathophysiology factors involved in vascular calcification and its genetic influence was performed. *Methods.* A systematic review was conducted in the Medline and PubMed databases and were searched for studies concerning vascular calcification using the keywords and studies published until 2020/01 in English. *Inclusion Criteria*. Studies in vitro, animal models, and humans. These include cohort (both retrospective and prospective cohort studies), case-control, cross-sectional, and systematic reviews. *Exclusion Criteria*. Studies before 2003 of the existing literature.

## 1. Introduction

According to data from the World Health Organization, there was a 17% decline in mortality from 2005 to 2015. Currently, the prevalence of cardiovascular diseases (CDs) remains high, 7.4% for men and 5.3% for women, due to the highest prevalence of cardiovascular risk factors (CRFs) and aging [1].

Recent evidence shows that there is a high prevalence of vascular calcification (VC) in atherosclerosis, diabetes, and chronic kidney disease, standing out as an independent CRF of morbidity and mortality [2, 3]. Data from the MESA study show that the calcium score (CAC), the main marker of CV, was associated with a gradual increase in risk events. In the multivariate analysis, considering the traditional risk factors, CAC >300 was associated with a nearly 7-fold increased risk (CI 95% 2.93–15.99) for cardiovascular outcomes compared to those with a zero CAC [4].

Depending on the histological arterial site, different types of VC can be distinguished: intimal calcification or medial artery calcification.

Intimal calcification is associated with atherosclerotic plaque formation comprises fibrous cap, necrotic/lipid nucleus, cholesterol crystals, inflammatory cells that are prone to erosion, rupture and obstruction of blood flow, reduced organ perfusion, and ischemic syndromes [5, 6].

Medial artery calcification, also called Mönckeberg calcification, represents the pathological deposition of calcium and phosphate in the medial layer of the arteries. This vascular calcification is considered less directly associated with inflammation. It can cause stiffness of the vascular wall, leading to changed arterial hemodynamic properties. This results in an increased chance of hypertension and left ventricular hypertrophy and aggravates the risk of cardiac events [5, 6].

In the past, VC was considered being the consequence of a passive degenerative process, but recent studies suggest the existence of a complex physiopathological mechanism still in elucidation that involves inflammation, oxidation, and release of chemical mediators. This mediator promotes or inhibits differentiation of vascular smooth muscle cells (VSMCs) in osteo/chondroblastic cells. There is also evidence of genetic factors involved in this process [7, 8].

**Figure 1:**
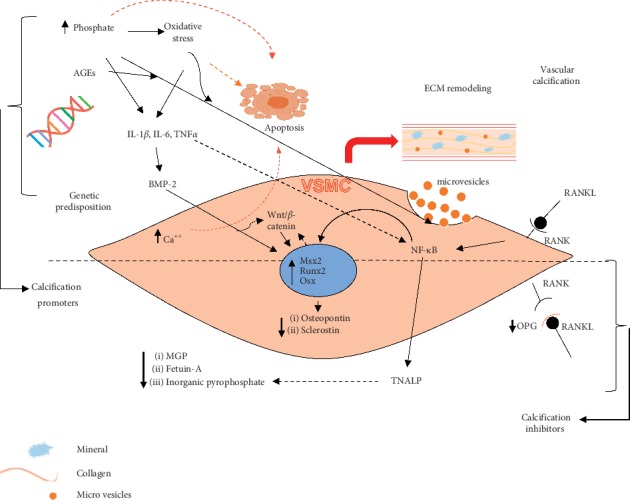
Pathophysiological mechanisms which promote cellular differentiation and vascular calcification. AGEs, advanced glycation end products; VSMC, vascular smooth muscle cell; BMP, bone morphogenetic protein; Runx2, runt-related transcription factor 2; Msx2, msh homeobox 2; IL-1*β*, interleukin-1beta; IL-6, interleukin-6; TNF*α*, tumor necrosis factor alpha; NF-*κ*B, nuclear factor kappa-light-chain-enhancer of activated B cells; RANK, receptor activator of nuclear factor kappa-B; RANKL, receptor activator of nuclear factor kappa-B ligand; ECM, extracellular matrix; OPG, osteoprotegerin; MGP, matrix gla protein; TNALP, tissue nonspecific alkaline phosphatase.

In this review, we will appraise the main cellular mechanisms, chemical mediators, and genetic components that involve the pathophysiology of vascular calcification (Figure 1).

## 2. Pathophysiology

### 2.1. Vascular Calcification Inducing Factors Inflammation, Oxidative Stress, and Lipids

It is recognized that VC is associated with a chronic and low-grade inflammatory state, exacerbated by nonvascular conditions such as metabolic alterations, autoimmune diseases, environmental factors (pollution), cancer, and obesity [7–10].

This inflammatory process was related to the increase of serum levels of TNF-alpha IL-1b promoting the stimulation of tissue nonspecific alkaline phosphatase (TNAP) activity, the enzyme that hydrolyzes pyrophosphate ions, which are potent inhibitors of mineralization and, therefore, can induce calcification regardless of the main transcription factor for osteoblasts RUNX2, in VSMC [7, 11]. Moreover, IL-6 was significantly associated with coronary artery calcification and cardiovascular mortality in patients with impaired renal function [12].

Studies have reported that increased oxidative stress is related to VC in vitro; recently, Zhao et al. showed that mitochondrial reactive oxygen species (ROS) are the major source of ROS production and are essential for the Pi-triggered phenotype switch of SMCs from a contractile to an osteogenic form [13].

Proinflammatory proteins include C-reactive protein (CRP) synthesized during inflammatory processes mainly by hepatocytes. Also, they are expressed by other cell types including local production in vascular tissue by VSMCs [14]. An in vitro study discloses a novel direct role of CRP during vascular calcification. CRP induces cellular oxidative stress and activates procalcific intracellular signaling pathways promoting osteo/chondrogenic transdifferentiation of VSMCs in vitro by the increased expression of TNAP and activity of the enzyme Fc fragment of IgG receptor IIa [15].

Oxidized LDL (oxLDL) plays a crucial role in the development of VC. The suggested mechanism is a reduction of prostacyclin production and the reduction of cyclooxygenase expression induced by the sustained increase of intracellular calcium. Those changes lead to the rupture of the glycocalyx monolayer integrity that causes endothelial cell apoptosis and the inflammatory process [16].

HDL plays a protective role in atherosclerosis by taking part in the process known as reverse cholesterol transport. In this process, it is believed that the existence of anti-inflammatory and antioxidant properties, probably dependent on the proteins, transported in the HDL particle, such as the enzyme paraoxonase and the reduction of cytokines with consequent attenuation of the osteogenic differentiation induced by oxidized LDL in vascular cells [17]. However, it was observed that patients with established coronary artery disease could have dysfunctional HDL production caused by posttranslational modification of apoA or by the reduction of HDL production induced by microRNAs [18].

### 2.2. Extracellular Vesicles: Exosomas

Extracellular vesicles are derived from different kinds of cells and classified into 3 classes by origin and size: (1) microvesicles (100–500 nm), which originate from cell membrane evaginations, (2) exosomes (40–100 nm), which have the origin of intracellular organelles, and (3) apoptotic bodies (∼1,000 nm), which are generated during programmed cell death [19].

It has been suggested that, during cellular osteochondrogenic differentiation, exosomes are released by VSMC from intracellular multivesicular bodies induced by the production of sphingomyelin phosphodiesterase 3 in response to environmental stress of calcium [20]. The serine protein encoded by the SORT1 gene is associated also with vascular calcification. It was localized in humans and mouse atheromas promoting in vitro the formation of microcalcifications and extracellular vesicles stimulating the alkaline phosphatase release [21].

Other factors associated with exosome formation are the annexins (Anx 6) in the VSMC and Anx 5 in the macrophages that comprise a class of calcium-dependent proteins that mediate cellular processes such as exocytosis and endocytosis. They also participate in regulating inflammation, coagulation, and fibrinolysis [22–24].

### 2.3. Runt-Related Transcription Factor 2 (Runx2)

RUNX2 is the main transcription factor in vascular calcification, and its activity is subjected to regulation by phosphorylation events mediated by kinases and by nuclear translocation, DNA-binding capacity, and interaction with transcriptional cofactors. Runx2 regulates the expression of genes related to osteoblasts, such as osterix, receptor activator of nuclear factor kappa-*β* ligand (RANKL), and type I collagen [25–28].

Tang et al. showed that cells isolated from the middle layer of the vessel wall are expressed as markers Sox17, Sox10, S100*β*, and neural medium filament polypeptide and differentiated into CML-like cells and later into osteoblastic cells [29]. In an animal model, with SM22-Cre mice with Runx2 exon 8 floxed, the inhibition of receptor activator of nuclear factor kappa-*β* ligand (RANKL) was observed. RANKL was associated with decreased macrophage infiltration and formation of osteoclast-like cells in the aortic wall [30]. However, in other work using Runx2 knockout mice, no reduction in RANKL expression, macrophage infiltration, or atherosclerotic lesion size was observed compared to the control group. Instead, it was found a decrease in the lesion mineralization, besides the substantially decreased expression of osteocalcin, alkaline phosphatase, and chondrocyte maturation [31].

### 2.4. Bone Morphogenetic Proteins (BMPs)

BMPs are a group of proteins expressed by myofibroblasts, and they belong to the family of transforming growth factor-beta. BMPs are known for their important roles during embryogenesis and in the maintenance and repair of bones and other tissues in adults. The most known one is the BMP2 that has osteogenic activities that were related to oxidative stress, inflammation, and hyperglycemia [32]. The mechanism of stimulation would be mediated by the expression of Runx2 and by the induction of apoptosis of vascular smooth muscle cells, an event that starts vascular calcification [33]. BMPs also bind to type II and type I serine-threonine kinase receptors (bone morphogenetic protein receptor-IA (BMPRIA), BMPR-IB, activin receptor-like kinase-2 (ALK-2), and ALK1) to form complexes that regulate the phosphorylation of Smad1/5/8 and then combine with Smad4 protein. They together translocate to the nucleus where they are involved in the transcription of genes related to osteoblast differentiation, including ERK (extracellular regulated by signal kinase), and JNK (protein c-Jun N-terminal kinase) [34].

In contrast to BMP2, BMP 7 had a cytoprotective action for vascular proliferative disorders. In a coronary, carotid, and abdominal aorta in a diabetes-enriched cohort with 920 subjects, the SNPs rs6127984, rs6123674, and rs6123678 of BMP7 were independently associated with lower VC [35]. This antagonistic action could be mediated by the existence of specific receptors, such as endoglin (a type III TGF receptor), which binds to BMP-2 and not to BMP-7 [33].

### 2.5. Osteocalcin (OC)

OC are vitamin K-dependent proteins, expressed by preosteoclasts and osteoclasts. Total OC includes both carboxylated osteocalcin (cOC), which has a high affinity for hydroxyapatite, located predominantly in the bone matrix, and undercarboxylated osteocalcin (ucOC), which represents between 40 and 60% of the total circulating osteocalcin. This protein was recently associated with metabolic and cardiovascular disorders [36, 37]. The role of OC in VC is controversial. A recent inflammatory protocol, demonstrated using interferon‐*γ* and tumor necrosis factor‐*α*, was used to examine the acute (24 hr) and chronic (144 hr) effects of ucOCN in subcultured human aortic endothelial cells (HAECs) and VSMCs. The ucOCN did not affect inflammatory cytokine production, nor inflammatory signaling pathways in VSMCs or HAECs [38]. In another study, Saad et al. observed in elderly patients with metabolic syndrome that osteocalcin serum levels were negatively correlated with carotid atherosclerosis [37]. However, other works suggest the opposite idea.

An animal model with cultured C57BL/6 thoracic aorta revealed that calcification was correlated with increased expression of osteocalcin [39]. It is suggested that the mechanism by which OC promotes SMC differentiation and mineralization is mediated by the production of Sox9, Runx2, collagen type X, and proteoglycans and stimulating glucose metabolism in vascular cells via hypoxia-inducible factor 1*α* (HIF-1*α*) [40]. Also, Flammer et al. showed that high levels of CO were strongly associated with unstable CAD [41].

### 2.6. Receptor Activator of Nuclear Factor Kappa-B/ Receptor Activator of Nuclear Factor Kappa-B Ligand (RANK/ RANKL)

Growing evidence suggests that the binomial of RANK/RANKL may be important players in vascular calcification. RANK is present in atherosclerotic plaques and valvular heart disease. It is a type I membrane protein expressed on the surface of osteoclasts and is involved in their activation upon ligand (RANKL) binding. RANKL is a transmembrane protein, but a soluble form (soluble RANKL is sRANKL) also circulates in the blood. RANKL binds as a homotrimer to RANK on target cells [42, 43].

It has been demonstrated that RANKL stimulates vascular calcification by binding to RANK through the alternative NF-*κ*B pathway. Also it was indicated that RANK indirectly promoted vascular smooth muscle cell calcification by enhancing macrophage paracrine procalcific activity. This is achieved through the release of IL6 and TNFa binding of TNF receptor-associated factors (TRAFs 2, 5, and 6) to specific sites in the cytoplasmic domain of RANK [42, 43].

### 2.7. Nuclear Factor-Kappa B (NF-*κ*B)

Nuclear factor-kappa B (NF-*κ*B) is an essential protein for cell proliferation and migration. The increase in NF-*κ*B expression in VC is mediated by multiple factors, the main one being TNF*α*, which decreases the expression of ankylosis protein homolog (ANKH). This is a transmembrane protein that controls the efflux of pyrophosphate cells [44]. Voelkl et al. showed that in vitro and in animal model under conditions of calcification, the serum- and glucocorticoid-inducible kinase 1 (SGK1) are powerful regulators of NF-*κ*B activity [45]. Studies have also shown that the advanced glycation end products (AGEs) and their receptors (RAGEs) are involved in this process [46, 47].

NF-*κ*B is a DNA-binding protein that contributes to the process of vascular calcification by multiple mechanisms. It triggers the transcription of TNF*α*, IL1*β*, and IL6, enables the action of the RANKL/RANK pathway, and can modulate the proinflammatory cascade in VSMCs when associated with the WNT/*β*-catenin signaling pathway in response to hyperphosphatemia. It also promotes the calcification of VSMCs by the expression of msh homeobox 2 (MSX2) and, consequently, Runx2. Also, it increases the expression of tristetraprolin (TTP), a destabilizing RNA protein, and reduces the ANKH mRNA decreasing pyrophosphate levels in the extracellular space [47, 48].

### 2.8. Canonical Signaling Pathway: Wingless/Beta Catenin (Wnt/*β* Catenin)

The Wnt/*β* catenin pathway is involved in a variety of physiological processes including tissue/organ differentiation, morphogenesis, and in many aspects in the development and progression of vascular lesions. This vascular injury includes endothelial dysfunction, macrophage activation, proliferation, and vascular smooth muscle cell migration. In adults, these glycoproteins take part of the key developmental and physiological processes, including cell proliferation, differentiation, migration, and apoptosis [48, 49].

The activation of the Wnt/*β*-catenin pathway is the binding of a Wnt ligand from the extracellular environment to two transmembrane proteins: a Frizzled protein acting as the receptor and a low-density lipoprotein receptor-related protein 5 or 6 (Lrp5/6) acting as a coreceptor [50]. It is established that this pathway is essential for the VSMC differentiation [51].

The Wnt/*β*-catenin release is enhanced by the action of different mechanisms [25]. We can mention Msx2 suppresses the antagonist Dkk1 that interacts with the LRP5/6 coreceptor [52].

WNT/*β*-catenin acts by stimulating the production of Runx2, the main CV factor, and consequently the Sp7 transcription factor (osterix) during hyperphosphatemia [53]. Also, it can participate even more in the calcification by induction of MMP2 and MMP9 in VSMCs and thus plays a crucial role in the progression of this phenomenon. [54].

### 2.9. Pentraxin 3 (PTX3)

Pentraxin 3 (PTX3) is a member of the pentraxin family. It is produced primarily in the liver and vascular endothelial cells in response to TNF*α*, IL-1, and IL-6 [52–54]. It is also released by peripheral blood leukocytes, myeloid dendritic cells, smooth muscle cells, fibroblasts, adipocytes, chondrocytes, mesangial, and epithelial cells. It has been reported that PTX3 induces the expression of RANKL by human osteoblasts, promoting osteoclastogenesis in vitro culture system [52–55].

In a population-based study with apparently healthy adults from four ethnic groups, PTX3 was associated with CVD risk factors, subclinical CVD, CAC, and clinical CHD events. These associations were independent of CRP supporting the hypothesis that PTX3 reflects different aspects of atherosclerosis-related inflammation than CRP [56].

### 2.10. Vascular Calcification-Inhibiting Factors: Inorganic Pyrophosphate

Inorganic pyrophosphate (PPi) is the main known calcification inhibitor found in extracellular space [60]. The PPi is produced in VSMCs and transported from the intracellular to the extracellular environment by the membrane protein ANK (progressive ankylosis or ANKH) [25]. It also is produced in the liver through ATP-binding cassette subfamily C member 6 (ABCC6) protein [57].

PPi is hydrolyzed by ecto-nucleotide pyrophosphatase/phosphodiesterases (ENPP1) and local tissue-nonspecific alkaline phosphatase (TNAP) promoting pathophysiological vascular calcification [58, 59]. High extracellular phosphate, which is frequently present in CKD, diabetes, and aortic calcification, reduces the production of PPi by increasing the expression of ENPP1 and TNAP by inducing apoptosis of the VSMCs [25–60]. However, in other work, it was observed in experimental models that PPi synthesis increases in VSMCs during phosphate-induced calcification because of compensatory regulation of PPi extracellular metabolism [60].

### 2.11. Matrix Gla Protein (MGP)

Matrix gla protein (MGP) is a protein phosphorylated and carboxylated by vitamin K. It is synthesized in various cellular types (mesenchymal, vascular, and chondrocytes). The expression of the MGP gene can be regulated via various mechanisms that have the potential to become genomic biomarkers for the prediction of vascular calcification (VC) progression [61, 62].

MGP acts as a potent inhibitor of VC. The mechanism of action includes direct inhibition of calcium-phosphate precipitation, the formation of matrix vesicles (MVs), the formation of apoptotic bodies, and differentiation of VSMCs [55]. The specific action of MGP could be related to the functional inhibition of BMP 2 and BMP-4, which have a similar structure [62, 63].

### 2.12. Fetuin-A (F-A)

Strong evidence indicates that human vascular smooth muscle cells exposed to changes in the extracellular calcium and phosphorus concentration undergo phenotypic differentiation leading to calcification, especially in chronic kidney disease (CKD) [60, 64].

F-A is a plasma glycoprotein, which in adults is synthesized by the liver, is a circulating proteinaceous calcification inhibitor, being able to bind ∼100 Ca^2+^ ions per molecule. When F-A is exposed to high calcium and phosphate concentrations, the molecules coalesce to form the primary calciprotein particles (CPPs) that contain amorphous calcium phosphate with a diameter of 50–100 nm, and F-A keeps these particles in solution and prevents it from precipitation [47–65].

Observational studies have shown that serum F-A reduction was associated with higher overall mortality and CV in patients with CRD [65, 66]. Mice deficient in F-A presented soft tissue calcification when treated with diets enriched with vitamin D or phosphorus. In a study with subjects without diabetes and without renal dysfunction undergoing cardiac catheterization, multivariate logistic regression analysis revealed that F-A levels were inversely correlated with the presence of coronary calcification (OR: 0.54, 95% CI, *P* = 0.025). Similarly, low serum F-A levels were associated with greater severity of CD in a large multiethnic population without clinical CVD, regardless of traditional CV risk factors, gender, ethnicity, and renal function [67, 68].

The polymorphisms in the corresponding *α*2-Heremans-Schmid glycoprotein gene (AHSG) were shown to be associated with F-A serum levels. In the Diabetes Heart Study, SNPs of this molecule were analyzed in 829 diabetic adults; after multivariate analysis, two SNPs in AHSG in the exon region 6-7, rs2593813 and rs2070632, showed a significant association with CAC [69]. However, in a recent study, the molecular analysis of the AHSG T256S gene variant (rs4918) was performed when individuals with zero CAC to 10 and those with CAC >10 were compared, and there was no significant association with F-A [70].

### 2.13. Osteoprotegerin

OPG is a cytokine produced in the bone marrow derived from stromal cells. It acts on bone remodeling by inhibiting the binding of RANK to RANKL on the surface of osteoclasts and T lymphocytes [71]. The role in vascular calcification is not completely defined.

A protective action was proposed for the accelerated evolution of atherosclerosis in OPG knockout mice [72]. However, another study showed that a high level of OPG was associated with a moderate calcium score is high in diabetic individuals [73]. Findings suggest that OPG can be used as a disease marker, increasing the levels of this protein as a compensatory response to the loss of bone mass and vascular damage [74].

In a prospective study of 130 patients, Pessaro et al. showed that elevated RANKL levels were associated with CHD (OR 1.75 (95% CI 1.04–2.94, *P* = 0.035) [75]. Recently, Choe et al. analyzed genetic variations using SNPs representative of OPG (rs2073618), RANK (rs1805034), and RANKL (rs2073618) and investigated the association with atherosclerotic plaque instability assessed by cardiac catheterization. It was found that the non-TT genotype of rs9594782 RANKL SNP was an independent risk factor for the occurrence of acute coronary syndrome [76].

### 2.14. Dickkopf Proteins (Dkk)

The Dkk members include Dkk 1, 2, 3, and 4, among which only Dkk 1 is considered inhibitors of the canonical pathway Wnt/B-catenin, by blocking its interaction with coreceptors 5 and 6 of LDL (LRP5/6) [77]. Studies showed that Dkk-1 attenuates the expression of Runx2, which is a canonical Wnt target and an important transcription factor for the osteogenic transdifferentiation of VSMCs [53]. It has been suggested that high levels of Dkk-1 may contribute to the formation of unstable plaque by inhibiting the deposition of diffuse protective calcifications, in addition to influencing the cellular composition of the plaque [78].

Some studies related elevated serum levels of Dkk1 and the presence of VC by CAC [79]. Levels of Dkk-1 were also independently associated with a composite of cardiovascular death, myocardial infarction, or stroke and with cardiovascular death alone [80]. Fang et al. showed, in diabetic mice, with induced kidney disease, that the combination of Dkk1 neutralization and reduction of urinary phosphate by a phosphate ligand was sufficient to decrease vascular calcification [63].

### 2.15. Sclerostin

Protein that has been discovered in genetic studies of rare sclerosing bone dysplasias in humans is encoded by the SOST gene [81]. It is a 190 amino acid glycoprotein, a member of DAN proteins. Its structure consists of four different cysteine bonds synthesized by osteocytes with their gene expression also produced in the kidneys, liver, placenta, and cartilage. It binds to LRP (low-density lipoprotein receptor) 5 and 6 of the osteoblasts, inhibiting the Wnt pathway, which is a group of proteins that function as the genetic signaling of cell proliferation and the increase of osteoblastic activity and with antagonistic activity to BMP in the bone [81].

In vitro, sclerostin was expressed in the calcification process of vascular smooth muscle cells [74]. Recently, Nguyen-Yamamoto et al. showed in a mice model study that sclerostin protected the organism from VC by suppressing BMP2 production [82].

Elevated serum levels of this protein in individuals with chronic kidney disease were associated with a lower mortality rate, which leads to the hypothesis that sclerostin elevation is related to the protective function against vascular calcification [83].

### 2.16. Osteopontin (OP)

The role of OP in vascular calcification is still controversial; it is a protein that acts by binding to osteoclasts through avB3 integrin and stimulates calcium reabsorptive activity. It was found in calcified vascular tissue. In an experimental model, the protein inhibits the mineralization of vascular smooth muscle cells by direct binding to calcium crystals [84, 85]. However, in a study by Benezin et al., 126 subjects with type 2 DM and asymptomatic coronary artery disease, as determined by the CAC, the OP was an independent predictor of coronary calcification (OR = 3.23, 95% CI = 1.09–5.20; *P* = 0.044) [86].

### 2.17. Genetics

In addition to chemical mediators, genetic characteristics and predisposition also play an important role in VC (Table 1).

A genome-wide association study (GWAS) reported a total of 31 DCV-associated loci at wide genomic significance (*P* < 5 × 10^−8^), accounting for only 10% of CVD heritability. This result may be explained probably because of the polygenic nature of the locus identified [8].

Data from the CARDIoGRAMplusC4D Consortium study, with 63,746 cases and 130,681 controls, identified 15 loci of genomic significance. Initially, six loci had relevant cardiovascular phenotypes, namely, ABCG8, APOB, GUCY1A3, PLG, LPL, and FES. PLG is adjacent to LPA, and the risk variant of PLG rs4252120 is strongly associated with elevated Lp(a) lipoprotein levels. Of the 30 loci of susceptibility to CAD previously reported in individuals of European and South Asian descent, PEMT, APOE, LDLR, COL4A1, LIPA, APOA1 APOA5, PPAP2B, and PCSK9 also present phenotypic characteristics directly relevant to the disease [8].

Toutouzas et al. retrospectively evaluated coronary angiograms of patients with established CAD that were reinvestigated for stable/unstable angina. The −174C allele of the IL-6 gene has been shown to increase the risk of progression of coronary plaques over 4 years [65].

In another in vitro study, there was an overexpression of the cannabinoid receptor type 2 gene (CNR2), which is an inflammatory marker in atherosclerotic plaques, compared to normal arteries, but not exclusively in vulnerable plaques [66].

In a meta-analysis of broad exomic association, 25,109 European ancestors and African ancestors with CD and 52,869 participants using CAC and carotid medial-intimal thickening measured by ultrasonography found protein-coding variants in APOE *ε*2. They were significantly associated with subclinical and clinical atherosclerosis, independently of ethnicity (odds ratio 0.77, *P* = 1 × 10^−11^) [87].

Chasman et al., in a cross-sectional study with white individuals, a polygenic risk score derived from an analysis of 50 SNPs that had genomic significance for association with coronary artery disease in previous studies and lifestyle were associated with CAC. The standardized CAC was 46 Agatston units (95% CI, 39 to 54) among participants with high genetic risk, compared to CAC 21 Agatston units (95% CI, 18 to 25) among those with low genetic risk (*P* < 0.001). CAC was significantly higher among participants with an unfavorable lifestyle than among those with a favorable lifestyle: CAC 46 Agatston units (95% CI, 40 to 53) versus CAC 28 Agatston units (95% CI, 25 to 31) (*P* < 0.001) [88].

A recent systematic review of the literature with twelve studies showed that ADAMTS7 polymorphisms, especially SNP rs4380028 allele, have been consistently associated with CAD. ADAMTS7 rsrs4380028 was consistently related to coronary artery disease: both coronary artery calcification and coronary artery stenosis [89].

Van Setten et al. calculated polygenic scores based on SNPs with *P* values reported by the CARDIoGRAMplusC4D2 consortium that reached a predefined threshold (<5 × 10^−7^, <5 × 10^−6^, <5 × 10^−5^, <5 × 10^−4^, <5 × 10^−3^, <0.05, <0.1, <0.2, <0.3, <0.4, and <0.5), which resulted in 11 polygenic models containing between 39 and 15 475 SNPs [8]. Later, they performed the association with EC in 2599 participants of the Dutch and Belgian Lung Cancer Screening (NELSON) trial. This polygenic model could explain up to 13.9% of the variance observed in CAC. This suggests that a substantial fraction of the hereditary risk for CAD/MI is mediated by arterial calcification. Besides, it was observed that genetic variants associated with serum lipid levels and the body mass index influence the level of CAC [90].

Mendelian disorders are related to vascular calcification. For example, in genome-wide association, studies were able to identify loci at 6p21.3, 10q21.3, and 9p2 associated with CAC. It was also showed that ENPP1that encodes a cell surface protein that regulates extracellular phosphate is the gene responsible for idiopathic infantile arterial calcification (IIAC) [91].

Recently, mutations in the ABCC6 gene encoding a transmembrane ATP-binding protein were described in 14 of 28 children with IIAC and pseudoxanthoma elasticum. An animal model suggests that the ABCC6 gene is related to ectopic and vascular calcification. This calcification was attributed to reduced levels of *γ*-carboxylation of matrix g1a protein (MGP), an important calcification inhibitor [91].

## 3. Conclusion

The VC has a complex pathophysiology still under investigation. The knowledge of the relationship of genetic factors with this process certainly becomes important, not only for the field of basic research but also for better definition of progression, prognosis, and provision of new therapies in the prevention and treatment of atherosclerosis. Future studies should better elucidate the mechanism involved in the pathophysiology and genetics of vascular calcification processes.

## Figures and Tables

**Table 1:** Summary of vascular calcification mediators and their potential functions in promoting calcification.

Protein	Gene	Function	Calcification
IL-1*β*	IL1B (interleukin 1 beta)	Proinflammatory cytokine which promotes vascular calcification via NF-*κ*B and Wnt signaling pathway	Intima/media
IL-6	IL6 (interleukin 6)	Proinflammatory cytokine which promotes vascular calcification through inflammation-induced oxidative stress.	Intima/media
TNF*α* (tumor necrosis factor alpha)	TNF (tumor necrosis factor)	Inflammatory cytokine which promotes vascular calcification by increasing the expression of osteogenic genes.	Intima/media
CRP	CRP (C-reactive protein)	Proinflammatory protein may contribute to vascular calcification through the raised expression of osteogenic factors such as Runx2 and TNAP.	Intima/media
TNAP (tissue nonspecific alkaline phosphatase)	ALPL (gene encoding human TNAP)	Degrades inorganic pyrophosphate which makes VSMCs susceptible to calcification	Media
Cbfa1/RUNX2 (RUNX family transcription factor 2)	RUNX2 (RUNX family transcription factor 2)	Transcription factor involved in chondrocyte and osteoblast differentiation	Media
Osterix	SP7 (Sp7 transcription factor)	Controls the osteoblast differentiation and bone formation	Media
BMP-2 (bone morphogenetic protein 2)	BMP2 (bone morphogenetic protein 2)	Osteogenic and osteoblast proliferation factor which upregulates the expression of Runx2	Media
Osteocalcin	BGLAP (bone gamma-carboxyglutamate protein)	Protein derived from osteoblasts, which stimulates the PI3K/Akt signaling pathway and upregulating the nuclear factor-kappa *β* (NF-*кβ*)	Media
RANKL (receptor activator of nuclear factor kappa-B ligand)>	TNFS11 (TNF superfamily member)	Regulates osteoclast differentiation and activation	Media/intima
RANK (receptor activator of nuclear factor kappa-B)	TNFRSF11A (TNF receptor superfamily member 11a)	Activates the transcription factor NF-*κ*B for the generation and survival of osteoclasts	Media/intima
NF-*κ*B	NFKB (NF-kappa-B protein complex: NFKB1, NFKB2, RELA, RELB, and REL)	Transcription factor which promotes vascular calcification through the expression of MSX2 (which increases TNAP expression) and reduces the expression of ANKH through the expression of tritetraproline	Media/intima
Wnt/*β*-catenin	WNT1/CTNNB1 (Wnt family member 1/catenin beta 1)	Through its canonical pathway promotes osteogenic gene expression via nuclear *β*-catenin dependent transcription	Media
PTX3	PTX3 (pentraxin 3)	It has been reported that PTX3 induces the expression of RANKL by human osteoblasts, thereby promoting osteoclastogenesis in vitro culture system	Media
Inorganic pyrophosphate	—	Prevents the nucleation of amorphous calcium phosphate and inhibits hydroxyapatite crystallization	Media
ANKH	ANKH (ankylosis, progressive homolog)	Inhibits mineralization by exporting inorganic pyrophosphate to the extracellular space to inhibit hydroxyapatite formation	Media
Matrix gla protein	MGP (matrix gla protein)	Inhibits vascular mineralization by binding to calcium ions	Media
Fetuin A	AHSG (alpha 2-HS glycoprotein)	Inhibitor of calcification by binding to calcium ions and forming calciprotein particles (CCPs) which prevent the precipitation of amorphous calcium phosphate particles	Media
Osteoprotegerin	TNFRSF11B (TNF receptor superfamily member 11b)	Binds to RANKL as a decoy receptor resulting in down regulation of osteoclast differentiation, which decreases osteoclast activity	Media/intima
DKK1 (Dickkopf Wnt signaling pathway inhibitor 1)	DKK1 (Dickkopf Wnt signaling pathway inhibitor 1)	Inhibitor of the canonical *β*-catenin-dependent Wnt pathway, it was shown to reduce the expression of Runx2 which is a canonical Wnt target	Media
LRP5/6 (LDL receptor-related protein 5/6)	LRP5/6 (LDL receptor-related protein 5/6)	Wnt coreceptors that upon activation lead to stabilization of cytoplasm *β*-catenin, nuclear translocation, and target gene expression	Media
Sclerostin	SOST (sclerostin)	Inhibitor of Wnt/*β*-catenin signaling, decreases bone formation by inhibiting osteoblastogenesis, in osteoclasts, and enhances bone resorption	Media
Osteopontin	SPP1 (secreted phosphoprotein 1)	Inhibitor of mineralization and ectopic calcification linking the extracellular matrix with calcium	Media

## Abbreviations

CRF: Cardiovascular risk factor
VSMC: Vascular smooth muscle cell
CD: Cardiovascular disease
VC: Vascular calcification
CAC: Coronary artery calcium
CI: Confidence interval
IL: Interleukin
IIAC: Idiopathic infantile arterial calcification
TNF: Tumor necrosis factor
oxLDL: Oxidized low-density lipoprotein
HDL: High-density lipoprotein
Anx: Annexin
F-A: Fetuin-A
Cbfa/Runx2: Core-binding factor subunit 1 alpha/runt-related transcription factor 2
RCP: C-reactive protein
RANKL: Receptor activator of nuclear factor kappa-*β* ligand
BMPs: Bone morphogenetic proteins
MVs: Matrix vesicles
OC: Osteocalcin
OP: Osteopontin
OPG/RANK/RANKL: Osteoprotegerin system-nuclear factor receptor kappa-B ligand
PTX 3: Pentraxin 3
Wnt/*β* Catenin: Canonical signaling pathway Wingless/beta catenin
DKK: Dickkopf proteins
GWAS: Genome-wide association study
Lp: Lipoprotein
CNR2: Cannabinoid receptor type 2 gene
SNPs: Single nucleotide polymorphisms.
